# Compressor pulsation noise attenuation using reactive silencer with various configurations: A theoretical and experimental study

**DOI:** 10.1016/j.heliyon.2024.e27263

**Published:** 2024-02-29

**Authors:** Negar Rahmani, Yasaman Khastavan, Ali Safari Variani, Saeid Ahmadi

**Affiliations:** a Master of Occupational Health Engineering, School of Health, Qazvin University of Medical Sciences, Qazvin, Iran; b MSc of Occupational Health Engineering, School of Health, Qazvin University of Medical Sciences, Qazvin, Iran; c Department of Occupational Health and Safety, School of Health, Qazvin University of Medical Sciences, Qazvin, Iran

**Keywords:** Transmission loss, Expansion chamber silencer, COMSOL, Impedance tube

## Abstract

Compressors are a significant source of noise in various industries. Silencers can be utilized to mitigate this noise. This study aims to design and construct an expansion silencer that can effectively reduce the pulsating noise produced by a reciprocating compressor. This study employed a model-experimental approach to investigate the performance of four different sizes of expansion silencers in controlling the pulsating noise in the suction part of the compressor. Initially, the silencers' sound transmission loss and pressure loss were simulated using the finite element method with COMSOL software. Subsequently, the sound transmission loss of the silencers was measured according to the E261109 standard using an impedance tube. Finally, the pressure loss of the silencers was measured using a Pitot tube upstream and downstream of the silencer at various flow rates. The results of the modeling showed that increasing the diameter of the silencer leads to an increase in transmission loss at all frequencies. Additionally, raising the length of the silencer only increased the number of sound transmission loss peaks in the frequency bandwidth without significant change in sound transmission loss. Furthermore, the results of the experimental measurements with an impedance tube revealed that increasing the diameter results in increased transmission loss, while increasing the silencer length leads to an increase in the number of transmission loss peaks without altering the transmission loss. Moreover, the modeling and experimental pressure loss results indicated that increasing the diameter of the expansion chamber causes an increase in pressure loss, while increasing the length of the expansion chamber results in a minor change in pressure loss. Finally, the research results showed relatively good agreement between modeling and experimental outcomes.

## 1 Introduction

An air compressor is a pneumatic device that converts power, such as an electric motor, diesel, or gasoline engine, into potential energy stored in compressed air. The compressor automatically shuts off once the reservoir pressure reaches its predetermined limit. The compressed air remains in the tank until needed (“Air compressor. 2020,”). Generally, compressors or densifiers are utilized to compress fluids that can be compressed. In specific devices and machines, air is compressed by compressors and then directed to the combustion section [1]. Compressors are also widely used in various applications, including household appliances like refrigerators, freezers, air conditioners, and vacuum cleaners, as well as in medical equipment such as dental drills and hospital air supply. The aviation industry relies on compressors to provide compressed air for turbine engines, while industries utilize compressed air for pneumatic systems, gas condensate, and gas storage. Moreover, compressors are essential components in air conditioning systems, vehicles, and other industrial machines, each with unique working mechanisms [2]. Among the various positive displacement compressors, reciprocating compressors, also known as oil piston compressors, hold significant importance as they have been used in industrial and domestic settings for many years [1]. These compressors generate noise during operation due to structural vibrations and flow turbulence. The primary sources of noise production in compressors are the working mechanism, pressure fluctuations, valve operation, gas movement within the pipes, belt movement, vibrations in the discharge pipe, suspension system, lubrication, and body design [3,4]. In a reciprocating compressor, there is typically a pulsating airflow. As a result of the pulsating air flow, 'pulsating noise' is also generated. In a reciprocating compressor, the component responsible for the suction process yields the most pronounced pulsating or throbbing noise [1,3].

Given the widespread use of compressors, it is evident that noise control for this equipment is of utmost importance [5]. In general, noise control in sound production sources can be achieved through two methods: active noise control and passive noise control. Passive noise control methods are preferred over active ones due to their smaller geometries, lower costs, and better adaptation to harsh environmental conditions [6,7]. Enclosures and silencers are commonly used among the various passive noise control methods. Therefore, to effectively control the passive sound produced by piston, centrifugal, axial, and other types of compressors, enclosure, and silencer methods can be employed [8,9]. Silencers minimize the noise produced by various sources, including internal combustion engines, fans, compressors, turbines, air conditioning systems, and sources that generate aerodynamic noise from exhaust gas or air [10]. Generally, silencers can be categorized into reactive, dissipative, and hybrid types. Reactive silencers generate dissipative sound waves using geometric discontinuity and acoustics impedance difference [11].

A simple expansion muffler is known as a reactive muffler and is used to reduce low-frequency octave band noise. This type of silencer is used for applications where pulsed sound is generated from the gas flow, such as engine suction, exhaust, and compressor. The mechanism by which this silencer reduces sound transmission is by reflecting acoustic energy back to the source, thereby diminishing the overall sound transmission [12,13]. The Determination of the sound transmission loss^1^ is facilitated by modeling, as it does not depend on the properties of the sound source and the flow characteristics. One of the most widely used methods for calculating sound transmission loss is the finite element method used for numerical research [14,15]. This method is used to understand how an object behaves under different physical conditions [16,17]. Moreover, impedance tube, which is one of the standard methods for determining the sound transmission loss of silencers, can be used to accurately determine the sound transmission loss [18].

The simple expansion silencer primarily provides a sound reduction in the low-frequency range (500 Hz and below) and has the most significant impact. Additionally, to effectively reduce sound within a narrow range of the frequency spectrum, the use of reactive silencers, particularly the expansion type, is recommended. The sound reduction function in reactive silencers is periodic, meaning they have the highest sound transmission loss at specific frequencies and the slightest sound transmission loss at others. It is important to note that piston compressors predominantly produce low-frequency sounds. Therefore, using expansion silencers can effectively control the sound generated by piston compressors [19,20].

The selection and design of the silencer are crucial factors in determining the level of noise reduction[21]. The effectiveness of a simple expansion silencer in reducing sound is heavily influenced by its geometry. It is essential to carefully select the length and diameter of a reactive silencer to achieve optimal sound performance[22,23]. Another essential consideration in silencer design is the amount of back pressure or pressure loss that occurs when installed in the air or gas flow path, which can result in power loss for the system. The ideal design for a silencer is one that not only reduces sound pressure levels but also minimizes back pressure and pressure loss [23]. The primary cause of the pressure loss in a muffler is the friction of airflow within the interior channel, as well as the pressure changes resulting from variations in the cross-sectional structure of the channel, such as inflections. These changes in flow state led to local changes in air pressure, contributing to the overall pressure loss. Both of these factors result from the fluid movement overcoming viscous shearing stress, which includes local losses. The pressure loss is primarily attributed to partial loss and the reconfiguration of the vortex region and velocity within the local structure[24].

This study aimed to design and manufacture an expansion silencer with optimal length and diameter to control the pulsating sound generated by piston compressors effectively. The sound transmission loss of 10 dB was considered a reference for designing a simple expansion silencer. First, a length of 17 cm and a diameter of 3.7 cm were calculated to achieve this value. Furthermore, our research aimed to assess and compare the effectiveness of various muffler structures in terms of sound transmission loss, pressure loss, and other relevant parameters to design an optimal silencer. Since the length and diameter of silencers are crucial factors in the optimal muffler design, different dimensions were used to evaluate the performance of silencers.

Current research also aimed to mitigate supplementary expenses by analyzing and comparing various aspects of silencers. Therefore, by utilizing the outcomes of this study, designers can significantly minimize additional costs and enhance efficiency across different parts. In addition, this study explored the design of the most suitable dimensions for the silencer to minimize pressure loss. An ideal silencer design reduces the sound pressure and minimizes back pressure and pressure loss. What’s more is that, this study used modeling and experimental methods for validation, which has been undertaken in only a few studies.

## 2 Materials and methods

Measurement and frequency analysis of compressor suction noise were performed, and the results were modeled using COMSOL software^2^ to design, manufacture, and evaluate expansion silencer performance. A sound level meter, calibrator, anemometer, alcohol glass thermometer, pressure gauge, pitot tube, and impedance tube were used in the measurement and analysis phases.

The pressure level and sound power of the pulsating noise of the suction pipe of an oil piston compressor were measured with a sound level meter. Frequency analysis of the sound was measured and analyzed at points A and Z at 10 points on the hemisphere relative to the end surface of the suction pipe. A sound transmission loss of 10 dB was used as a reference for designing a simple expansion silencer based on the target frequency. The target frequency was determined based on the measurement results, and then the value of n was calculated. The optimum length of the expansion chamber is calculated based on one-quarter of the wavelength of the target frequency to achieve maximum sound transmission loss [[25], [26], [27]].

After drawing the silencers in the COMSOL software environment, COMSOL Multiphysics 5.4 was used to measure the transmission loss and pressure loss concerning the acoustic and aerodynamic performance of the silencers. Four different silencer geometries were modeled in 3D using the software during acoustic modeling. Using the modified Helmholtz equation, the models were analyzed for sound pressure in the frequency domain [28].

**Fig. 1:**
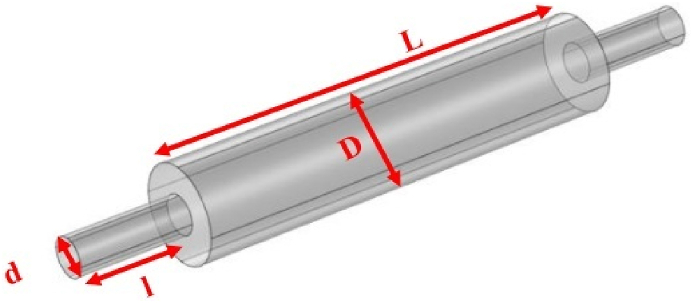
Expansion silencer with the diameter of the expansion chamber 3.7 cm and the length of the expansion chamber 17 cm.

**Fig. 2:**
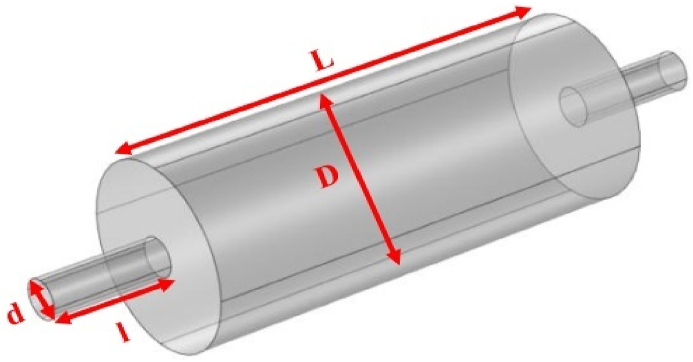
Expansion silencer with the diameter of the expansion chamber 7 cm and the length of the expansion chamber 17 cm.

**Fig. 3:**
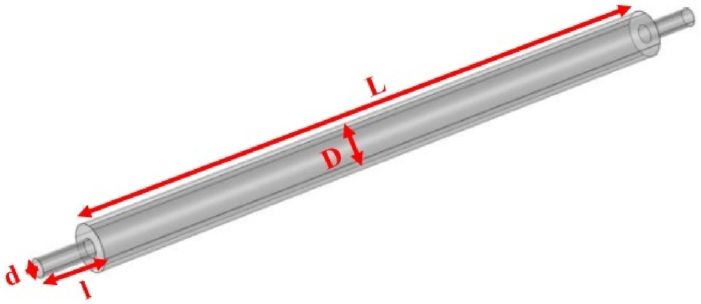
Expansion silencer with the diameter of the expansion chamber 3.7 cm and the length of the expansion chamber 51 cm.

**Fig. 4:**
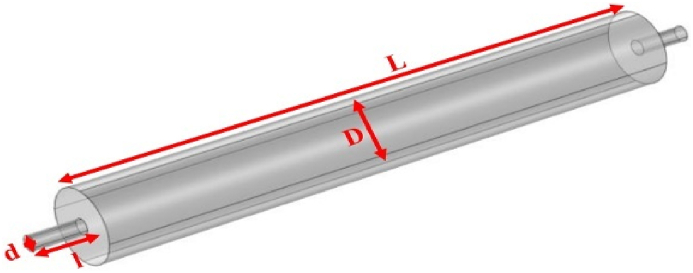
Expansion silencer with the diameter of the expansion chamber 7 cm and the length of the expansion chamber 51 cm.

Modeling was performed for four different silencer geometries with diameters of 3.7 and 7 cm and 17 and 51 cm lengths, as shown in Fig. 1, Fig. 2, Fig. 3, Fig. 4 The silencer geometries were designed as cylinders with central inlet and outlet tubes at the end of the cylinder with a diameter of D and a length of L. The different shapes correspond to the geometry required for acoustic analysis when connected to the impedance tube. The influence of the geometry of the expansion silencer has been studied in different models.

**Fig. 5:**
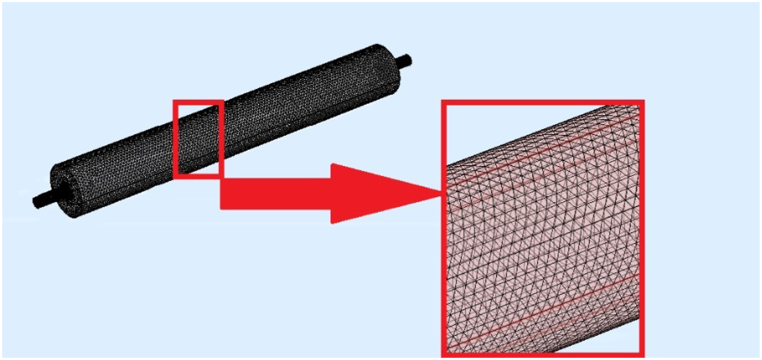
The mesh used to evaluate the acoustic part of the silencer.

The sound transmission loss is one of the most critical parameters for acoustic analysis of silencers and indicates the effectiveness of sound reduction by the silencer. It is defined and calculated as the input and output sound energy ratio at different frequencies. The outer walls of the expansion chamber and the inlet and outlet pipes are considered solid surfaces of the silencer[29]. A tetrahedral mesh with a size of one-fifth of the highest frequency wavelength *λ*_max_/5 was used to mesh the silencer (see Fig. 5).

In the fluid dynamic modeling, a two-dimensional symmetric model with a steady flow was used to study the aerodynamic performance of the silencers[30,31]. The total pressure of the silencers was studied at points spaced five times the channel diameter in the upstream and downstream parts of the expansion chamber. At the inlet, upstream, and downstream of the silencer, the total pressure resulting from the different airflow velocities and, thus, the pressure loss due to the different silencer dimensions was studied.

The boundary conditions include the flow velocity at the inlet, the pressure at the outlet, and different wall types. The fluid used in this study is assumed to be an ideal gas, namely air. An automatic tetrahedral mesh was used to create a magnificent mesh. Iron pipes with different inner diameters and lengths were used in welding and cutting during the manufacture of silencers.

**Fig. 6:**
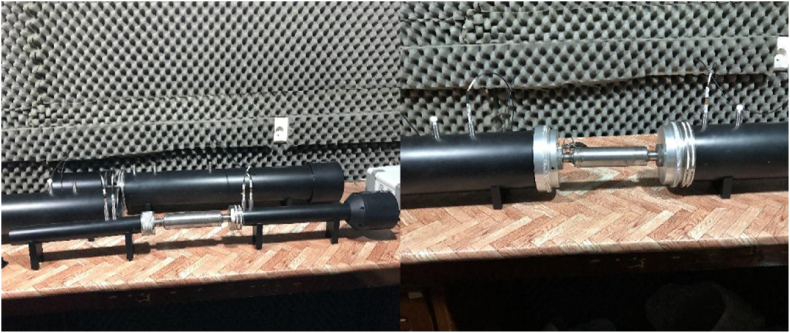
Measurement of sound transmission loss by impedance tube.

In order to evaluate the acoustic performance of the silencers, the sound transmission loss was measured with the impedance tube in an anechoic chamber (see Fig. 6). The sound transmission loss of expansion silencers was calculated using two impedance tubes with three and 10 cm diameters. The silencers were acoustically analyzed twice with a diameter of 3 cm and twice with a diameter of 10 cm. Three transmission loss curves in three frequency ranges were generated for each muffler, and the impedance tube modeling software then integrated these three curves to create the transmission loss curve of the studied silencers in the frequency bandwidth from 63 to 6300 Hz. In the impedance tube, the amplifier model PA50 and two software programs, VA-Lab 4 Basic and VA-Lab 4 IMP-AT, were used for the acoustic analysis of silencers; the microphones used in this impedance tube were MC3242.

**Fig. 7:**
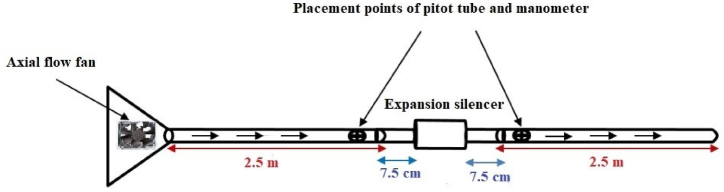
Schematic image of expansion silencer pressure loss measurement method.

An axial fan with model FFB0612EHE was used to measure the pressure loss in the silencers (Fig. 7). Different flow velocities were generated at the inlet and outlet of the muffler by changing the fan power. The total pressure was measured at a distance of five times the diameter of the channel from the silencer using a small pitot tube and a pressure gauge[32,33].

## 3 Result

### 3.1 Analysis of sound frequency around the suction pipe of a piston compressor

The results of the measurement and analysis of the sound frequency of the compressor suction pipe, performed at octave band frequencies on A-weighting and C-weighting networks, showed that the frequency of 500 Hz had the highest sound pressure level in both weighting networks and was therefore considered the dominant frequency. Additionally, a sound transmission loss of 10 dB at 500 Hz was used as a reference for designing a simple expansion silencer (Table 1).

**Table 1:** The results of the measurement and analysis of the sound frequency of the compressor suction pipe.

Frequency (Hz)	32	63	125	250	500	1000	2000	4000	8000	Total sound pressure level
Sound pressure level (dBZ)	61.1	66.9	63.8	69	73.3	65.5	61.3	52.3	46.9	77.6
Sound pressure level(dBA)	27.6	40.4	49.5	61.2	67.7	64.3	61	55.3	45	71.4

### 3.2 Sound transmission loss modeling for silencers

The cut-off frequencies for silencers with diameters of 3.7 and 7 cm and lengths of 17 and 51 cm were calculated to be 11623, 5978, 11623, and 5978 Hz, respectively. These calculations assumed plane waves were used for modeling, and a sound velocity of 343 m/s in air was considered. Table 2 shows the amount of sound transmission loss for all four silencers.

**Table 2:** The amount of sound transmission loss for all four silencers.

Silencer	Silencer sound transmission loss in the 500 Hz frequency band (dB)	Silencer sound transmission loss in the sound transmission loss curve and frequency of 500 Hz (dB)
Diameter 3.7 cm and length 17 cm	9.1	9.39
Diameter 7 cm and length 17 cm	20.3	20.7
Diameter 3.7 cm and length 51 cm	6.8	9.21
Diameter 7 cm and length 51 cm	17.6	20.48

### 3.3 Modeling sound transmission loss for the silencer in connection with an impedance tube

According to Table 3 the cutoff frequencies for the silencer with a diameter of 3.7 cm and lengths of 17 and 51 cm and for the silencer with a diameter of 7 cm and lengths of 17 and 51 cm, when connected to an impedance tube of 3 and 10 cm, are 11400 and 4221 Hz, respectively. Considering that the investigated frequency range is 1600 Hz when the silencer is connected to an impedance tube of diameter 3 cm and 6300 Hz when the impedance tube is connected to an impedance tube of diameter 10 cm, the investigated frequency is lower than the cut-off frequency. The cutoff frequencies for the silencer with a diameter of 7 cm and lengths of 17 and 51 cm, when connected to impedance tubes of 3 and 10 cm, were found to be 6030 and 4221 Hz, respectively. In both silencers, the studied frequency range during connection to an impedance tube with a diameter of 3 was 1600 Hz, and for an impedance tube with a diameter of 10, was 6300 Hz. The cutoff frequency within the studied frequency range was observed in the sound transmission loss curve. Consequently, the curves were gradual, with no sudden changes in sound transmission loss.

**Table 3:** Comparison of the cutoff and the studied frequency in all four silencers.

Silencer	Impedance tube diameter (cm)	Cutoff frequency (Hz)	The studied frequency (Hz)
Diameter 3.7 cm and length 17 cm	3	11400	1600
	10	4221	6300
Diameter 7 cm and length 17 cm	3	6030	1600
	10	4221	6300
Diameter 3.7 cm and Length 51 cm	3	11400	1600
	10	4221	6300
Diameter 7 cm and length 51 cm	3	6030	1600
	10	4221	6300

The silencers with diameters of 3.7 and 7 and lengths of 17 exhibited their maximum sound transmission loss at a frequency of approximately 1500 Hz, while the silencer with diameters of 3.7 and 7 and a length of 51 had its maximum sound transmission loss at a frequency of around 1300 Hz.

### 3.4 Comparison of silencer performance in modeling

When comparing the performance of silencers with different diameters but equal lengths (as shown in Fig. 1, Fig. 2), it becomes clear that increasing the diameter of the expansion chamber leads to an overall increase in sound transmission loss across all frequencies. In both cases, for expansion chamber diameters of 3.7 cm and 7 cm and lengths of 17 cm and 51 cm, respectively, 500 Hz and 125 Hz frequencies showed the maximum sound transmission loss (see Table 4).

**Table 4:** Transmission loss of expansion silencers in modeling.

Frequency (Hz)	Diameter 3.7 cm and Length 17 cm	Diameter 7 cm and Length 17 cm	Diameter 3.7 cm and Length 51 cm	Diameter 7 cm and Length 51 cm
32	0.5	4.6	2.9	12
63	1.3	8.1	5.6	16.2
125	3.6	13.1	8.6	19.9
250	7.1	18.1	6.5	17.3
500	9.1	20.3	6.8	17.6
1000	5.4	15.9	6.8	17.7
2000	7.6	18.8	6.9	18
4000	7.1	19.5	7.3	19.5
8000	4.7	12.2	4.3	16.8

Similarly, when comparing the performance of silencers with equal diameters but different lengths (as shown in Fig. 3, Fig. 4), it is evident that increasing the length of the expansion chamber resulted in an increase in the number of transmission loss peaks without significant change in sound transmission loss.

**Fig. 8:**
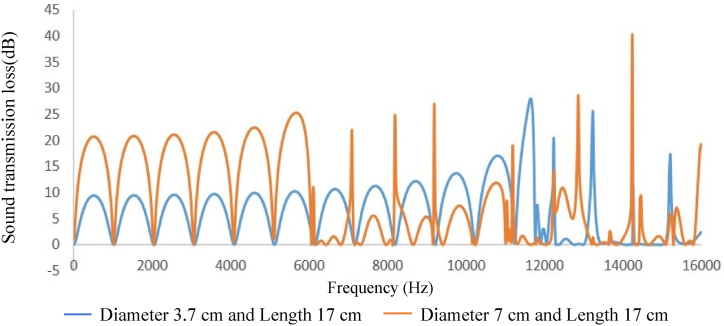
Expansion silencer transmission loss curve with length 17 and diameters 3.7 and 7 cm.

**Fig. 9:**
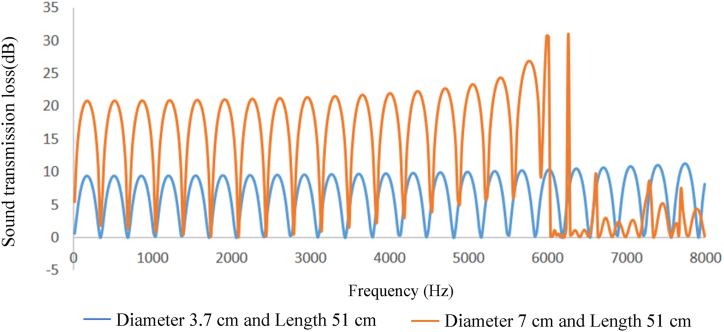
Expansion silencer transmission loss curve with length 51 and diameters 3.7 and 7 cm.

**Fig. 10:**
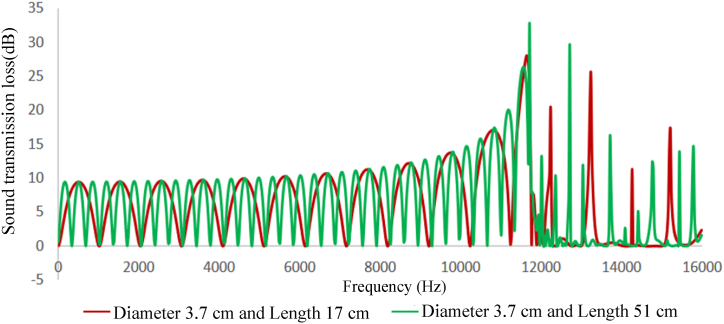
Expansion silencer transmission loss curve with a diameter of 3.7 and lengths of 17 and 51 cm.

**Fig. 11:**
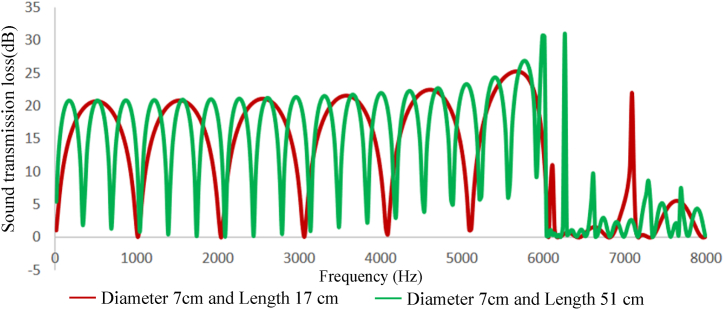
Expansion silencer transmission loss curve with a diameter of 7 and lengths of 17 and 51 cm.

In comparing silencer performance with different diameters and equal lengths, and vice versa, when connected to an impedance tube, it was observed that increasing the diameter of the expansion chamber led to an increase in sound transmission loss, while increasing the length of the expansion chamber resulted in an increase in the peak of the sound transmission loss curve and the amount of sound transmission loss did not change (see Fig. 8, Fig. 9, Fig. 10, Fig. 11).

### 3.5 Impedance transmission loss results - experimental

Table 3 compares the cutoff and studied frequencies modeled with an impedance tube. The maximum sound transmission loss was observed in silencers with diameters of 3.7 cm and 7 cm and lengths of 17 cm, with diameters of 7 cm and lengths of 51 cm, at frequencies of approximately 1500, 5500, 1300, and 5000 Hz, respectively.

Furthermore, comparing the silencers revealed that increasing the diameter also causes an increase in transmission loss, and increasing the silencer length causes an increase in transmission loss peaks.

### 3.6 Comparison of modeling and experimental results

**Fig. 12:**
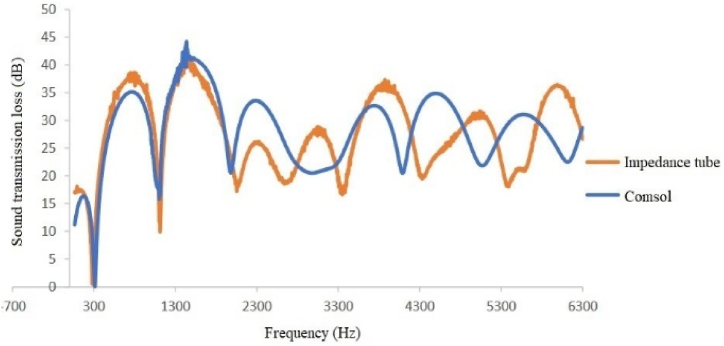
Comparison of the modeled sound transmission loss and the result of an impedance tube, expansion silencer with the diameter and length of the expansion chamber 3.7 and 17 cm.

**Fig. 13:**
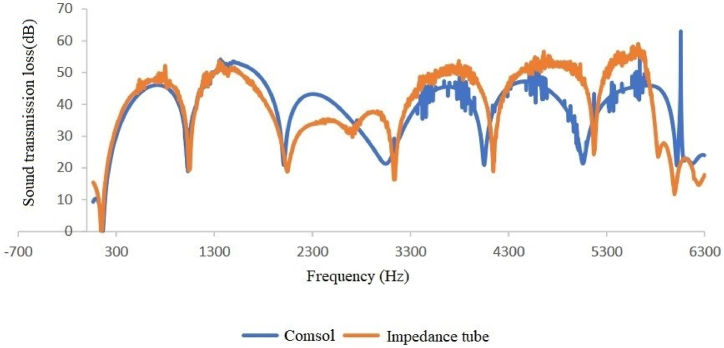
Comparison of the modeled sound transmission loss and the result of an impedance tube, expansion silencer with the diameter and length of the expansion chamber 7 and 17 cm.

**Fig. 14:**
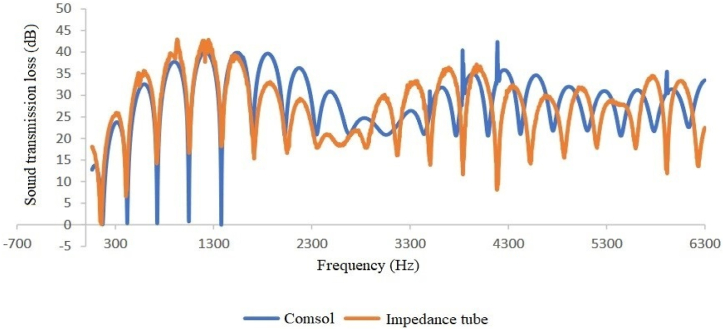
Comparison of the modeled sound transmission loss and the result of an impedance tube, expansion silencer with the diameter and length of the expansion chamber 3.7 and 51 cm.

**Fig. 15:**
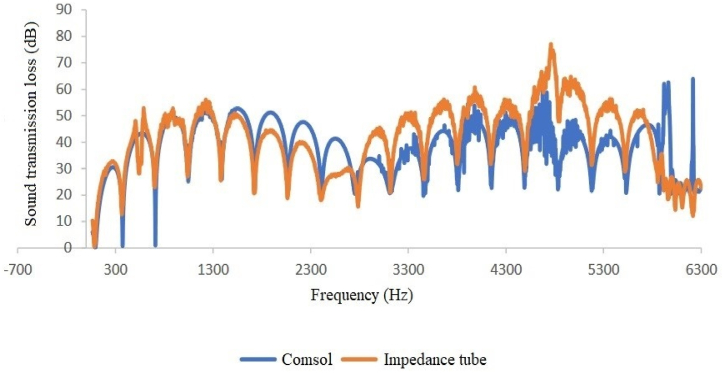
Comparison of the modeled sound transmission loss and the result of an impedance tube, expansion silencer with the diameter and length of the expansion chamber 7 and 51 cm.

A comparison of silencer transmission loss modeled and obtained through an impedance tube shows good agreement between experimental and modeling results at low frequencies (up to approximately 1600 Hz) in all four types of silencers (Fig. 12, Fig. 13, Fig. 14, Fig. 15). Furthermore, in two sound transmission loss curves in both the experimental and model cases, the maximum and minimum transmission loss values match at a specific frequency, and the curves are similar in shape at low frequencies.

However, in silencers with similar diameters of 7 cm but different lengths of 17 cm and 51 cm, the modeled values are slightly higher than the experimental values at middle frequencies. In contrast, the opposite is true at higher frequencies. Additionally, in the silencer with a diameter of 3.7 cm and a length of 51 cm, the modeled values are slightly higher than the experimental values at middle frequencies.

### 3.7 Silencer pressure loss results

The modeling and experimental pressure loss results indicate that, among the examined silencers, the silencer with a diameter of 3.7 cm and a length of 51 cm has the lowest pressure loss. In comparison, the silencer with a diameter of 7 cm and a length of 17 cm has the highest pressure loss. Specifically, at an inlet flow velocity of 4.5 m/s (when the fan operates at maximum power), the pressure loss for the silencer with a diameter of 3.7 cm and a length of 51 cm was 20.4 Pa. In contrast, the silencer, with a diameter of 7 cm and a length of 17 cm, was 26.7 Pa (see Table 5, Table 6).

**Table 5:** Modeling the pressure loss of silencers at different flow velocities with COMSOL.

Flow velocity	Diameter 3.7 cm and Length 17 cm	Diameter 7 cm and Length 17 cm	Diameter 3.7 cm and Length 51 cm	Diameter 7 cm and Length 51 cm
1	1.2	1.4	1.1	1.3
1.5	2.6	3.1	2.4	2.9
2	4.5	5.5	4.2	5
2.5	6.9	8.4	6.5	7.8
3	9.9	11.9	9.3	11.1
3.5	13.3	16	12.5	15
4	17.3	20.7	16.2	19.5
4.5	21.7	26.7	20.4	24.5

**Table 6:** Pressure loss of silencers at different flow velocities over experimental measurement.

Flow velocity	Diameter 3.7 cm and Length 17 cm	Diameter 7 cm and Length 17 cm	Diameter 3.7 cm and Length 51 cm	Diameter 7 cm and Length 51 cm
1	1.2	1.5	1.1	1.3
1.5	2.6	3.8	2.25	2.8
2	4.9	5.8	4.5	5.6
2.5	7.4	8.8	7.3	8.3
3	10.3	12.5	10	11.6
3.5	14	17.5	12.9	15.8
4	18.5	23	17	21
4.5	23.9	31	23	27.5

Both the experimental and modeled pressure loss results show that the silencer with a diameter of 3.7 cm and a length of 51 cm has the lowest pressure loss. In comparison, the silencer with a diameter of 7 cm and a length of 17 cm has the highest pressure loss, with a pressure loss of 23 Pa for the former and 33 Pa for the latter at an inlet flow velocity of 4.5 m/s.

**Fig. 16:**
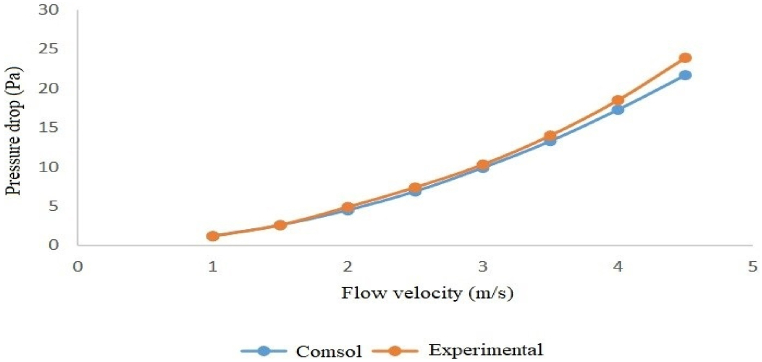
Modeled and measured pressure loss in the expansion silencer with the diameter and length of the expansion chamber 3.7 and 17 cm.

**Fig. 17:**
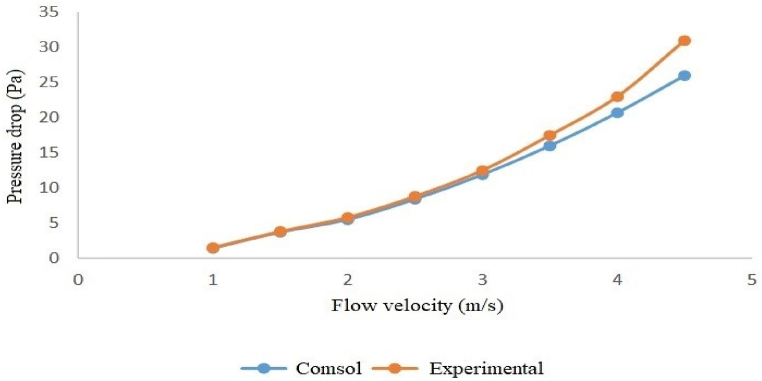
Modeled and measured pressure loss in the expansion silencer with the diameter and length of the expansion chamber 7 and 17 cm.

**Fig. 18:**
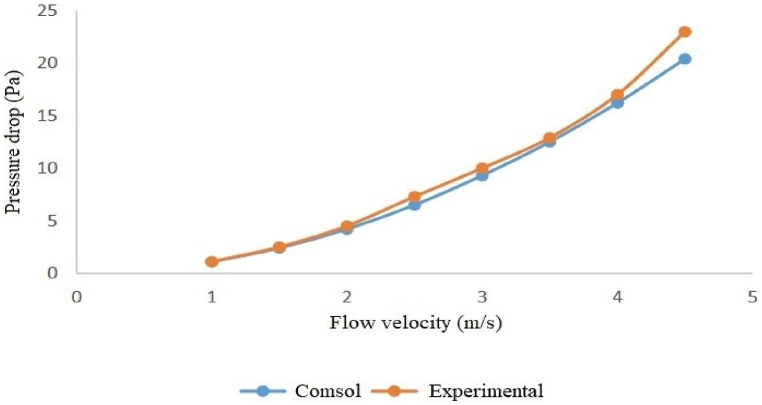
Modeled and measured pressure loss in the expansion silencer with the diameter and length of the expansion chamber 3.7 and 51 cm.

**Fig. 19:**
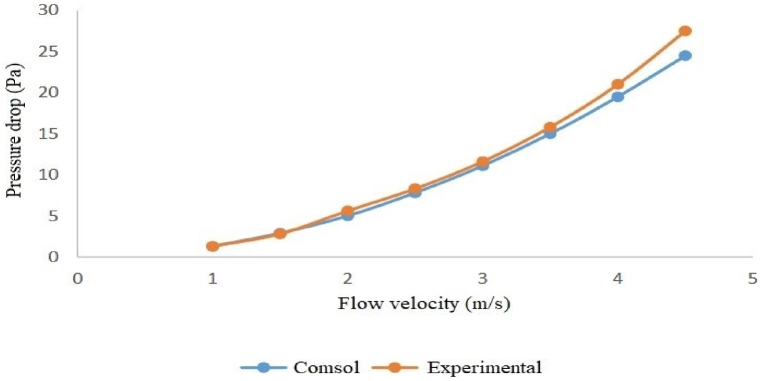
Modeled and measured pressure loss in the expansion silencer with the diameter and length of the expansion chamber 7 and 17 cm.

Furthermore, the graphs below illustrate that, for all four silencers, the pressure loss remains consistent at low velocities, but the results diverge slightly as the flow rate increases (Fig. 16, Fig. 17, Fig. 18, Fig. 19).

## 4 Discussion

This study investigated the performance of four silencers, including expansion silencers with diameters of 3.7 and 7 and lengths of 17 and 51 cm, to reduce compressor noise in both the modeling and experimental sections. The modeling section studied the silencers’ sound transmission loss and pressure loss using COMSOL software. In the experimental section, sound transmission loss in the case of connection to an impedance tube and pressure loss in silencers were measured and evaluated.

In modeling the sound transmission loss, a simple expansion silencer was designed based on theoretical calculations to achieve a sound transmission loss of 10 dB at a frequency of 500 Hz. In order to achieve this sound reduction, the diameter and length of the simple expansion silencer were calculated to be 3.7 and 17 cm, respectively. The modeling of an expansion silencer with a diameter of 3.7 cm and a length of 17 cm based on the finite element method and using the COMSOL software resulted in a transmission loss of 10 dB at a frequency of 500 Hz for this type of silencer. On the other hand, the predicted sound transmission loss curves were sinusoidal and accompanied by peaks and troughs consistent with previous studies in this field. The silencer was designed to achieve maximum sound transmission loss at the compressor noise’s dominant frequency (500 Hz). The results obtained from this study are as follows:

The results obtained from modeling silencers with different diameters demonstrate that the transmission loss becomes greater as the silencer diameter increases. This increase in transmission loss is due to the dependence of the acoustic resistance of a channel on its cross-sectional area. A sudden change in the cross-section of a channel causes an impedance mismatch, resulting in sound wave energy being returned to the source. The greater the cross-section area change, the greater the impedance mismatch. Therefore, in this study, as the diameter of the silencer increases, the amount of transmitted wave energy decreases due to the change in impedance. Additionally, comparing the cut-off frequencies of silencers with diameters of 3.7 cm and 7 cm reveals that as the diameter of the silencer increases, the cut-off frequency decreases. This leads to wave filtering with lower frequencies than silencers with smaller diameters. This can be observed in Fig. 8, Fig. 9, where the transmission loss curve of the silencer with a larger diameter is not a plane wave, and higher order modes are created from 5978 to 16000 Hz. On the other hand, in the silencer curve with a smaller diameter, the transmission loss curve from 11623 to 16000 Hz is not a plane wave, and higher order modes have been created. It should be noted that one-dimensional analyzes using modeling software that is based on plane waves can accurately predict the performance of silencers up to the cutoff frequency, and these analyzes have less accuracy at higher frequencies and complex geometries.

Furthermore, the outcomes of silencers modeling with varying lengths demonstrate that the number of peaks or the maximum transmission loss has raised with the increase in length. The graphs shown in Fig. 10, Fig. 11, which correspond to different lengths in the simulation, illustrate that within the frequency range of 32–11623 Hz, solely the transmission loss peaks have increased with the expansion chamber’s lengthening. Additionally, noting the equal diameters of silencers, the cut-off frequency remains consistent in both curves with varying lengths. Similar results were achieved in modeling the impedance tube-connected silencers, wherein an increase in diameter resulted in a greater transmission loss, and increasing the length led to a greater number of transmission loss peaks.

Finally, the research results showed relatively good agreement between modeling and experimental outcomes (Fig. 12, Fig. 13, Fig. 14, Fig. 15). Both results indicate that an increased diameter leads to higher transmission loss. Additionally, increasing the length only increases the number of transmission loss peaks without affecting the maximum value.

The study conducted by Zhaorong Zhang and Jianliang Li was consistent with this research. As the diameter of the expansion chamber increases, the sound transmission loss in all frequencies increases. With the increase in the length of the expansion chamber, only the peaks of the sound transmission loss in the frequency bandwidth increase [34].

The results of pressure loss modeling of the silencers using COMSOL software for different flow velocities showed that the pressure loss of the silencers also increases as the airflow through the silencers increases. The highest-pressure loss at all flow velocities was found for the silencer with a diameter of 7 and a length of 17 cm, and the lowest pressure loss was found for the silencer with a diameter of 3.7 and a length of 51 cm. At all flow rates, the silencers are ranked in terms of increase in pressure loss as follows: Silencers with a diameter of 3.7 and a length of 51 cm, silencers with a diameter of 3.7 and a length of 17 cm, Silencers with a diameter of 7 and a length of 51 cm, and Silencers with a diameter of 7 and a length of 17 cm. If the length of the expansion chamber in the silencer is constant, an increase in the diameter of the expansion chamber leads to a rise in the pressure loss. If the diameter of the expansion chamber is constant, an increase in the length of the silencer leads to a decrease in the pressure loss of the expansion silencer.

The results of the study conducted by Jum Fu et al. (2020) titled "Structural Parameters' Effects on the Acoustic Attenuation of Diesel Engine Silencer and Analysis of Prominent Structural Parameters” showed that changes in perforation rate, short axis ratio, and length to diameter ratio have a negligible effect on the acoustic attenuation of the silencer at a frequency of 2000 Hz. The performance of the silencer is more affected by changes in the length-to-diameter ratio and then the perforation rate concerning the short axis. The analysis of the sound pressure characteristics of the silencer has also shown that the sound pressure varies significantly in the high-frequency range(J. [35]).

The results of the study conducted by Jukka Tanttari et al. [36] titled "Increasing the Acoustic Attenuation of Microperforated Reactive Silencers” showed that for the short version of the silencer, the maximum simulated TL value was significantly higher than the maximum measured TL value. The maximum simulated and measured TL values better agree with the extended version. For both versions, the range of simulated TL is significantly more extensive than the range of measured TL. The reason for these differences is leakage between the rear chambers and sound generated by the structure itself[36].

The limitations of this research included the lack of a suitable space, such as a workshop for the construction and testing of studied silencers, and inadequate facilities and equipment. For instance, we didn't have enough equipment to measure the transmission loss of the silencer by the impedance tube, so we made a part to connect the input and output of the silencer to the impedance tube.

## 5 Conclusion

This paper investigated the acoustic performance of four silencers with different lengths and diameters through modeling and experimental tests. The sound transmission loss of 10 dB at a frequency of 500 Hz was considered as a reference for designing simple expansion silencers. Using COMSOL software and the finite element method, this sound transmission loss was predicted for a silencer with a diameter of 3.7 cm and a length of 17 cm. Results demonstrated that increasing the diameter of the silencer leads to the reflection of a greater portion of the input sound wave energy back to the sound source through impedance changes. Therefore, a larger sound transmission loss is achieved in silencers with bigger diameters. Furthermore, raising the length of the silencer only increased the number of sound transmission loss peaks in the frequency bandwidth, and the change in sound transmission loss was not noticeable. Additionally, increasing the silencer length only led to a minor change in pressure loss. Since the acoustic performance of the two silencers with different lengths was almost similar, it can be concluded that designers can achieve the same results by choosing a silencer with a smaller length to prevent additional costs for constructing such silencers.

## Data availability statement

The data that has been used is confidential.

## Additional information

No additional information is available for this paper.

## CRediT authorship contribution statement

**Negar Rahmani:** Software, Investigation, Formal analysis. **Yasaman Khastavan:** Writing – original draft. **Ali Safari Variani:** Resources, Formal analysis, Data curation. **Saeid Ahmadi:** Supervision, Project administration, Methodology, Formal analysis, Data curation.

## Declaration of competing interest

The authors declare that they have no known competing financial interests or personal relationships that could have appeared to influence the work reported in this paper.

## Footnotes

1 Sound transmission loss (STL) is a quantification of how much sound energy is prevented from traveling through an acoustic treatment.

2 COMSOL software is a finite element analysis, solver, and simulation software package for various physics and engineering applications, especially coupled phenomena and Multiphysics.
